# Auditory abilities of speakers who persisted, or recovered, from stuttering

**DOI:** 10.1016/j.jfludis.2006.07.001

**Published:** 2006

**Authors:** Peter Howell, Stephen Davis, Sheila M. Williams

**Affiliations:** Department of Psychology, University College London, Gower Street, London WC1E 6BT, England

**Keywords:** Persistent stuttering, Recovered stuttering, Hearing, Backward masking

## Abstract

**Objective:**

The purpose of this study was to see whether participants who persist in their stutter have poorer sensitivity in a backward masking task compared to those participants who recover from their stutter.

**Design:**

The auditory sensitivity of 30 children who stutter was tested on absolute threshold, simultaneous masking, backward masking with a broadband and with a notched noise masker. The participants had been seen and diagnosed as stuttering at least 1 year before their 12th birthday. The participants were assessed again at age 12 plus to establish whether their stutter had persisted or recovered. Persistence or recovery was based on participant's, parent's and researcher's assessment and Riley's [Riley, G. D. (1994). *Stuttering severity instrument for children and adults* (3rd ed.). Austin, TX: Pro-Ed.] Stuttering Severity Instrument-3. Based on this assessment, 12 speakers had persisted and 18 had recovered from stuttering.

**Results:**

Thresholds differed significantly between persistent and recovered groups for the broadband backward-masked stimulus (thresholds being higher for the persistent group).

**Conclusions:**

Backward masking performance at teenage is one factor that distinguishes speakers who persist in their stutter from those who recover.

***Education objectives***: Readers of this article should: (1) explain why auditory factors have been implicated in stuttering; (2) summarise the work that has examined whether peripheral, and/or central, hearing are problems in stuttering; (3) explain how the hearing ability of persistent and recovered stutterers may differ; (4) discuss how hearing disorders have been implicated in other language disorders.

Stuttering often starts in childhood, though the problem frequently remits before teenage. Statistics about recovery during childhood were given by Andrews et al. (1983). They analyzed results from several studies and estimated that 75% of those stuttering at age 4 years, 50% of those stuttering at age 6 years, and 25% of those stuttering at age 10 years, recovered by the time they reached 16 years of age. If the problem continues to around teenage, the chance of recovery decreases. Thus, Andrews and Harris's (1964) survey data show that no child who was stuttering when they passed age 12 years recovered by age 16 years. (The survey ceased when participants were around this age.)

Recovery and persistence of stuttering has been assessed in several different ways. For instance, Andrews and Harris (1964) used a population-based sample of all children born between May and June 1947 in Newcastle-on-Tyne in the United Kingdom. Initially, there were 1142 respondents. The study only located a small number of children who stuttered and this subsample is not considered adequate according to some authorities (Yairi & Ambrose, 2005). Also, audio recordings do not appear to have been made for their participants. Thus, clinicians seem to have made their speech-based assessments in real time. The approach of Yairi and Ambrose (2005) has been to locate speakers who are close to the onset of their stuttering and follow them up, typically to about age 8. The children are recorded and have been assessed on various language, motor and demographic instruments longitudinally. Members of this team are clinically trained. Diagnosis of stuttering in such very young children is not always easy, even for clinicians (as Yairi's group's own work shows). Also recovery is not complete by age 8 (Andrews et al., 1983), so it is possible that some participants who have not recovered by this age will do so subsequently (up to age 12 according to Andrews & Harris, 1964). Our own work examines children from as near to 8 years as is possible and re-examines them at the minimum age of 12 years. Ages at initial testing are partly determined by the clinical populations that are available. The things that commend studying children at these ages are: (1) that the test range extends before and after the age at which most recovery is complete, (2) there is a realistic expectation that children at these ages can perform in the procedures required for testing (such as those used for hearing assessment in the current study), and (3) the age range complements that of Yairi and co-workers so it provides information their study cannot (and conversely, their study provides information which ours cannot).

The present study examines the extent to which auditory functioning is predictive of recovery from stuttering. The current study examined whether teenage participants who recovered or persisted in the disorder, differed in their performance on a range of auditory tasks. In the remainder of this section, (1) the criteria used for classifying participants who stutter as recovered or persistent are outlined and (2) the reasons for thinking auditory performance might differ between the two subgroups of participants who stutter are presented.

A participant may be considered to have recovered from stuttering (recovered developmental stutterer, RDS) if he or she (1) has been diagnosed as stuttering in childhood, (2) but is regarded as fluent at age 12 (Andrews & Harris, 1964). A past history of stuttering can be established by personal report (Wingate, 1976). However, a more satisfactory way is to obtain an independent clinical assessment at an age before recovery has taken place (at least 1 year before assessment of recovery in the work reported below). Speech samples obtained at this earlier age provide an objective record of the speaker's previous status (Wingate, 2002). The samples need to be analyzed using a standardized measurement instrument designed to assess frequency and severity of stuttering (Riley, 1994). Recovery is generally considered to be associated with a reduction in the frequency and severity of stuttering (e.g. Ingham, 1984; Starkweather, 1993; Yairi, 1993, 1996). To establish any such reduction, additional speech samples need to be obtained and analyzed using the standardized measurement instrument again (Riley, 1994) when the child passes age 12 by which time recovery will have taken place if it was going to happen at all (Andrews & Harris, 1964). It is possible that other difficulties remain even when there is a reduction in characteristics of stuttering in the speech (in which case recovery is only partial). Examples of such characteristics are speech naturalness (Lees, 1994), and the overall effect of stuttering on the speaker's ability to communicate (Yaruss, 2001). These are characteristics that are determined during early clinical assessments, but these are not usually available at age 12, particularly for cases where speakers have recovered because they typically do not see their clinician around this age. These aspects of communication were also assessed at teenage: (a) by the children who stutter, (b) by their parents, and (c) by researchers.

A participant may be considered to persist in stuttering (persistent developmental stutterer, PDS) if he or she (1) has a past history of stuttering, and (2) is regarded as still stuttering at age 12. Participants who persist in their stuttering had to be considered to be stuttering by an independent clinician, and for this to be reflected in the analysis of the samples of their speech (Riley, 1994) at least 1 year before they passed age 12. They also had to have continuing difficulty at age 12 plus, based on analyses of their speech samples (Riley, 1994) and as assessed: (a) by the children who stutter, (b) by their parents, and (c) by researchers.

There are several lines of evidence that suggest that auditory processes may be involved in some way in stuttering. First, fluency control improves in participants who stutter if the sound of the voice is altered before the participant hears it. Various forms of noise maskers (Cherry & Sayers, 1956; Dewar, Dewar, Austin, & Brash, 1979), as well as frequency shifted (Howell, El-Yaniv, & Powell, 1987) and delayed (Ryan, 1974), versions of the voice, all improve control in participants who stutter. The improvements could be the result of a deficit in the auditory system whose effects are attenuated when each of these alterations is made. Second, there are a number of reports of physiological differences between speakers who stutter and fluent controls, some of which discuss the auditory cortex and its relationship to other cortical and subcortical regions. For example, Sommer, Koch, Paulus, Weiller, and Buchel (2002) reported decreased fractional anisotropy diffusion in white matter in speakers who persist in their stutter compared to controls. They interpreted this finding as showing decreased myelinisation below the left sensorimotor representation of the tongue and larynx. Also, in an anatomical study using MRI, Foundas, Bollich, Corey, Hurley, and Heilman (2001) reported abnormalities of size and asymmetry in speakers who persist in their stutter in the planum temporale. The focus of the current study is on auditory functioning assessed behaviorally.

Auditory masking paradigms are often used to assess participants’ hearing ability. A probe tone that is masked by a noise stimulus should not be distinguishable from a masking noise alone. The paradigms used to assess performance with masked sounds present two or more sounds (three are used in the work below), all of which have the masking sounds, but only one of which has the probe tone. Listeners are required to indicate which interval contains the sound with the probe tone. The test starts with the probe tone loud enough to be easily distinguishable from the masking sound. Over a series of trials, the level of the probe tone is reduced until listeners cannot hear the probe and have to guess. At this point, the level of the probe tone is around the threshold appropriate for that listener for the given noise masker level. Listeners are encouraged to guess when they are unsure, and when they do so, they will make a correct response by chance on some of the trials (approximately 50% of the times when two intervals are presented and 33% of the times when three intervals are presented). To ensure the correct response was not a lucky guess, the level of the probe tone is increased slightly to bring it above the threshold for detection, and the test repeated. After the threshold has been crossed by increasing and decreasing the level of the probe tone, the true threshold can be estimated. Thresholds have been traditionally estimated like this for no masker (absolute threshold) and with a variety of different maskers. One important type of masking stimulus is a broadband masking stimulus presented concurrent with the probe. This is called a simultaneous masker and performance with this stimulus reflects cochlear processes (Moore, 1982).

Masking stimuli consisting of a probe tone followed by the masking stimulus (backward-masked stimuli) are being extensively investigated at present. Interest in backward masking was prompted by the work of Tallal and Piercy (1973), which was intended to establish auditory involvement in another language disorder, specific language impairment (SLI). SLI shares with stuttering difficulty in some aspects of speech production including late language onset (Andrews & Harris, 1964), word-access difficulties (Howell & Sackin, 2001) and possible problems dealing with grammatically complex structures (Howell & Dworzynski, 2005), but also involves deficits in comprehension in children who appear to be unimpaired in other cognitive tasks. Tallal and Piercy (1973) proposed that SLI stems from difficulties in processing the temporal structure of sounds, which would affect speech and language ability. Wright et al. (1997) conducted auditory backward and simultaneous masking tests with SLI children. Consistent with the view of an auditory deficit involving processing of temporal structure, Wright et al. (1997) found that SLI children have higher backward masking thresholds, but similar simultaneous masking thresholds, compared with control children.

There are two studies where backward masking performance of participants who stutter has been compared with fluent controls. Howell, Rosen, Hannigan, and Rustin (2000) compared backward masking performance of stuttering and control participants aged between 8 years 1 month and 12 years 6 months. The participants who stutter had poorer backward masking performance compared with the fluent controls. In more extensive testing, Howell and Williams (2004) tested the auditory sensitivity of 37 participants who stutter and 44 participants who do not stutter, aged between 8 and 19 years in the following five listening conditions: (1) absolute threshold, (2) simultaneous masking, (3) backward masking, (4) notched backward masking, and (5) simple dichotic (simultaneous) masking. Howell and Williams (2004) found no deficit in children who stutter relative to fluent controls in backward masking performance, although there was some evidence that the thresholds changed during development at different rates for the two groups of participants. Part of the reason that no difference was found between the groups of participants could be that only PDS show a backward masking deficit whereas RDS operate like fluent controls, and that Howell et al.'s (2000) sample included more participants destined to persist in the disorder.

The hypothesis tested here is that PDS participants have a backward masking deficit compared with the RDS participants. Four auditory conditions were selected from Howell and Williams (2004). These were absolute threshold and simultaneous masked threshold as controls (neither Howell et al., 2000, nor Howell and Williams found differences between PWS and controls for these conditions, so no differences would be expected between PDS and RDS speakers) and two conditions that involved variants of backward masking stimuli. The auditory tests were made when the participants were aged between 12 and 17 years, i.e. at an age at which the designation as PDS or RDS could be made. Howell and Williams (2004) have shown that participants of these ages can perform the task.

In summary, PDS speakers’ and RDS speakers’ performance with backward masking stimuli was examined (backward masking performance is regarded as reflecting aspects of speech processing performance). The question examined was whether the PDS speakers alone show backward masking deficits. This may help resolve some contradictory indications in the literature as to whether speakers who stutter have backward masking deficits, and indicate whether performance with these stimuli is a sign of PDS.

## 1 Method

### 1.1 Participants

Thirty-two participants took part in the study, all of whom had English as a first language. Two males were dropped from the study because the criteria for diagnosing the cases as PDS or RDS were equivocal (see below for further details). Twenty-five of the remaining 30 participants were male and 5 were female. The participants first were seen when they were referred to a clinic specializing in the treatment of stuttering. They were assessed at this time and confirmed as stuttering (see below for details). They were reassessed a minimum of 13 months later (again see later for details) at which time a series of hearing tests was administered. At this time, the participants also were reexamined to establish whether or not they were still stuttering. The ages at initial assessment and at subsequent assessment are given in Table 1. The mean length of time these second assessments were made after the initial ones was 40.00 months for those subsequently identified as PDS and 45.11 months for those subsequently identified as RDS. The difference in elapsed time between the initial assessment and the reassessment for PDS and RDS was not significant by *t*-test (*t*(28) = .973, *p* = .339). At the time of hearing assessment, the participants were aged between 12 years 1 month and 16 years 9 months (mean age 14.04). Twelve were PDS and 18 were RDS according to the criteria given below. Seventeen of the 30 participants (8 PDS and 9 RDS) were included in the Howell and Williams (2004) study.

### 1.2 Initial assessment of stuttering

The participants who stutter were initially diagnosed by a speech-language pathologist who worked in a local health authority. They then were referred to a clinic that specialized in childhood stuttering which they attended within three months (on average) where the diagnosis was confirmed by a second speech-language pathologist. The participants who stutter were first seen as part of this study with their parents at the time of referral to the clinic. Here the parents received advice about how to manage stuttering (including how to cope with bullying or teasing). This advice was constant for all the participants and reported to be restricted to this attendance. Retrospective checks were made in cases of spontaneous recovery to ascertain when stuttering ceased (Ingham, 1976). This was used to check whether application of the treatment procedure could have been responsible for alleviating stuttering. No cases were reported where cessation was within 12 months of treatment.

Participants were assessed using Riley's (1994) Stuttering Severity Instrument, version three (SSI-3) when they attended clinic. During the assessment, interviews of about 20 min duration were recorded in a quiet room using a Sennheiser K6 microphone and Sony DAT recorder. The recording included a reading of a text and a sample of spontaneous speech containing a minimum of 200 syllables. The interview was subsequently used to assess the frequency and duration of stuttering and any associated physical concomitants. These were scored according to the guidelines specified in Riley (1994). The researcher was trained to use SSI-3 and had about 10 years’ experience of research on stuttering. All participants (PDS and RDS) scored 22 or higher on SSI-3. A score of 22 is between the 41st and 60th percentile and rated as moderate stuttering. Although SSI-3 is a measure of severity rather than a way of differentiating fluent speakers from speakers who stutter, it has been used for contrasting the fluency of speakers who stutter with fluent speakers in other studies (Arnold, Conture, & Ohde, 2005; Davis, Shisca, & Howell, in press). Details of individual participants are given in Table 1 (identifier, gender, designation as persistent or recovered, age and SSI at initial assessment, age and SSI at the time of hearing assessment).

### 1.3 Assessment of stuttering at time of hearing assessment

Each participant was seen a second time after they reached 12 years of age. At this age, they had to be unambiguously designated as PDS or RDS (using the criteria indicated below) by the participant, parent, researcher and using Riley's (1994) SSI-3 scores.

#### 1.3.1 Participants’ assessments

Assessments were based on Boberg and Kully's (1994) questionnaire that they used for establishing the impact of their therapy program on stuttering. This questionnaire assessed 15 attributes, some of which were specific to their treatment. Seven were directly applicable for the current assessment (2, 6, 9, 10, 11, 12, 14) and three more (3, 4 and 5) were combined into one further attribute. All eight resulting attributes were assessed by giving a statement to which participants chose a response that matched their view. Table 2 presents the questions, the scale endpoints for the scale and the corresponding question numbers from Boberg and Kully (1994).

The responses to the eight attributes were summed for each participant to give a score of between 8 and 40, with high scores indicating continuing concern regarding stuttering. Scores of 21 and higher were considered as a reflection of persistence. Scores below 21 were considered a reflection of recovery. The score of 21 represents an average response across the eight questions of between two (a negative indication) and three (a neutral indication). (See later for details of how this score was combined with the other assessments.)

#### 1.3.2 Parents’ assessments

Parents’ views about the fluency of their son or daughter were assessed on the same eight attributes as above. The statements given in Table 2 were changed to third person, referring to the participant. The responses were scored in the same way as with the participants. The speech performance questionnaires were completed by the parents and children at the time of the researcher's assessments.

#### 1.3.3 Researchers’ assessments

The researcher who made the initial SSI-3 assessments visited the participant's home and recorded an interview that lasted approximately 90 min. The researcher gave a rating that was designed to complement those of the participant and his or her parent and to reflect what therapists/pathologists report doing when assessing a client's response to treatment. During his visit, the interviewer talked with the parent and child about the child's speech problem and experience in clinic. He also sought their views about communication style and self confidence in a range of typical environments, including home, social gatherings with adults and children out of school and in school. Performance and experience in school was assessed in terms of inter-personal relationships with staff and other children (including bullying). General health issues were also examined, including frequent absence from school and childhood illnesses. In 24/30 cases, two researchers attended. In cases where there was a second researcher, each rated the participant independently on a 9-point scale. Ratings were never more than 2 scale points apart. Discrepancies were resolved by discussion. In the six remaining cases, the rating of the single researcher was used. A score of four or more was designated PDS and a score less than four was considered RDS.

### 1.4 Stuttering Severity Instrument

During the second visit, a 20-min assessment of the speech of the participant was recorded using a Sennheiser K6 microphone and Sony DAT recorder. This was scored using the SSI-3 as at initial assessment (Riley, 1994). Individual SSI-3 scores at the second assessment are given in Table 1. Participants with scores of 24 and above (60th percentile and above, classified as moderate, severe or very severe on SSI-3) were regarded as PDS. Participants with scores lower than 24 were regarded as RDS. The 24-point criterion equates to approximately 3–4% stuttered syllables in the speaking and reading tasks, an average disfluency length of .5–1 s and physical concomitants rated as “not noticeable unless looking for it” or “barely noticeable to the casual observer”.

The minimum SSI-3 score at initial assessment was 22. All RDS participants scored below this value at second assessment except one, who scored 22. All PDS participants scored higher than 22 at second assessment. The initial and subsequent assessments were examined retrospectively. The mean SSI-3 score at initial assessment for those subsequently identified as PDS was 31.50 (S.D. 5.47) and 27.56 (S.D. 4.72) for the RDS. The difference between the PDS and RDS group at initial assessment was marginally significant by *t*-test (*t*(28) = 2.106, *p* = .044). The mean SSI-3 score at teenage for those identified as PDS was 29.67 (S.D. 4.14) and 14.06 (S.D. 5.07) for the RDS. The difference between the PDS and RDS group at teenage was highly significant by *t*-test (*t*(28) = 8.862, *p* < .000). Thus, there is only slight evidence for a difference in SSI-3 scores between PDS and RDS at initial assessment but substantial evidence at the time of the later assessment.

### 1.5 Criteria for designating participants as recovered or persistent

The criteria for stuttering at intake (for both PDS and RDS) were an SSI-3 score of 22 or greater and a specialist clinician's diagnosis that the child was stuttering. To be designated PDS, SSI-3 at the time of the second assessment had to be greater than 24, and the parent, child and researcher had to designate the child as still stuttering. To be designated RDS, SSI-3 at the time of the second assessment had to be less than 24 (little or no stuttering), and the parent, child and researcher had to designate the child as not stuttering. Out of the 32 participants originally seen, 2 participants (5.88%) were given responses by parents that were slightly at variance with the remaining assessments. In both cases, these were in the direction that the parents considered that there was less improvement than indicated by the other assessments. These were designated “unclassified” and these are the two cases whose data were not included in the study.

### 1.6 Equipment and stimuli

The stimulus for the hearing tests consisted of a brief probe tone that was not masked in one condition (absolute threshold) but was masked in one of three different ways in the remaining conditions. The probe tone was a 1-kHz sine wave, 20 ms in duration, including 10 ms raised cosine onset and offset gradients. The masker was a 300 ms band-limited white noise (Hartmann, 1979) with a spectrum level of 40 dB re 10^−12^ watts per Hz. The masker frequency range was 600–1400 Hz representing a 1.16 octave-wide band centered at 916 Hz. The temporal and spectral aspects of the experimental conditions were as follows:

(a) absolute threshold: 20-ms probe tone, no masker (masker level set at −15 dB spectrum level);
(b) simultaneous: 20-ms probe tone presented at a delay of 200 ms after onset of a 300 ms burst of the masker;
(c) backward: 20-ms probe tone presented immediately before a 300 ms burst of the masker;
(d) notched backward: the 20-ms probe tone immediately preceded a 300 ms burst of the masker, which had a notch in the masker between 800 and 1200 Hz.

All signals were generated and presented on a PC. Sound was output at 44.1 kHz through a Soundblaster 16-bit Plug and Play card via an MTR HPA-2 stereo headphone amplifier to Sennheiser HD250 linear 2 headphones. Level was changed by adjusting the digital waveform prior to D-to-A conversion. Headphone output was calibrated by playing a 1 kHz calibration tone (1960 ms in duration that had 10 ms raised cosine rise and fall) into a 6 cc coupler. The level of the tone was 79 dB SPL. Level in the coupler was adjusted to this value as measured with a type 2203 Brüel and Kjær SPL meter and type 4144 microphone cartridge. Sounds were presented in stereo throughout. Two output channels, each containing appropriate combinations of probe and masker signals for the selected condition, were passed to the amplifier and presented at the same amplification to each ear. The experiment was run in an AVTEC Amplisilence double-walled acoustic chamber.

### 1.7 Procedures

All hearing tests were conducted using a three-alternative forced choice (3AFC) procedure where participants indicated the interval in which they thought the probe was present.

For all the hearing tests, three faces were displayed on the computer screen, each of which changed from a neutral to an open-mouthed expression when its sound was presented (going in sequence from left to right). After the three sounds were presented, the participant indicated which of the three sounds had contained the stimulus by selecting the corresponding face graphic with the mouse-operated cursor and clicking on it. Feedback was given by an appropriate change in the selected graphic (smile or frown).

The computer randomized the presentation of the target sound between the three possible positions and collected, recorded, and evaluated the responses using a Levitt (1971) two down one up tracking procedure. There were 10 reversals per track and signal level adjustment was in 2 dB steps (the maximum resolution of the apparatus). The threshold estimate was the average of the last four reversals. Participants completed the hearing tests in a 1-h session. In this they completed the four different conditions, and threshold within these four conditions was evaluated up to three times each. The participant was re-tested if the difference between the first two evaluations was more than 2 dB.

## 2 Results

### 2.1 Backward masking performance of participants who have recovered or who persist in stuttering

Threshold estimates with a standard deviation greater than 5 dB over the last four reversals in the tracking algorithm, and all evaluations where the spread of threshold estimates exceeded 10 dB for the same condition, were examined and any for which there were obvious failures in the tracking were discarded. Failures included evaluations with multiple errors at starting levels which prevented the algorithm from working, and failures at levels that had been correctly identified repeatedly elsewhere in the same tracking evaluation and/or cases where the last two reversal pairs were in very different ranges. The latter typically gave rise to large standard deviations. A total of 285 evaluations were made for all 120 subject-by-stimulus-condition pairings (30 participants × 4 stimulus conditions). Threshold estimates were based on two evaluations in the majority of cases, but 45 required a third evaluation. Fourteen of the 285 evaluations had to be discarded (4.9% which compared with 2.3% in Howell & Williams, 2004). All remaining data were included in the analysis.

**Fig. 1:**
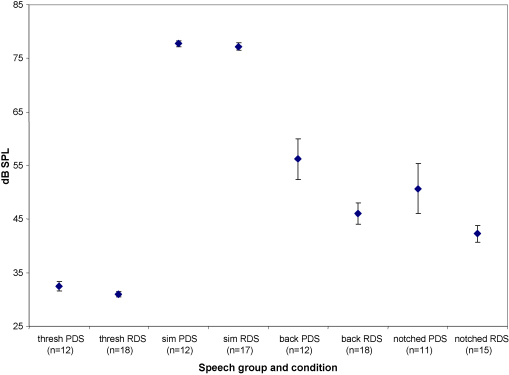
Mean threshold estimates and ±1 S.D. (labeled dB SPL on the ordinate) for persistent and recovered speakers who stutter for each stimulus condition. The stimulus conditions are absolute threshold, simultaneous masking, backward masking and notched noise backward masking (labeled thresh, sim, back and notched on the abscissa). The threshold estimates are shown separately for the persistent and recovered groups (labeled PDS and RDS, respectively). The numbers of participants where an estimate was obtained are indicated for each speaker group at each stimulus condition on the abscissa.

The threshold estimate for each participant for any particular stimulus condition was the average value of the remaining evaluations for that condition. It was not possible to obtain an estimate for the notched backward masking stimulus condition for one of the PDS participants and three of the RDS participants (indicated on the abscissa of Fig. 1) because all evaluations in these conditions were discarded. The only other condition where an estimate could not be obtained for a stimulus condition was for the simultaneous masking condition of an RDS participant.

The mean scores and ±1 S.D. for the PDS and RDS groups for each stimulus condition are given in Fig. 1. Independent *t*-tests were carried out on the performance measures for the four stimulus conditions. No correction was made for multiple comparisons, as the comparisons were planned and the number of comparisons was small (four) (Keppel, Saufley, & Tokunaga, 1992). The only condition where there was a significant difference between PDS and RDS participants was in the backward masking condition (*t*(28) = 2.579, *p* = .015), that for the notched backward masking condition was *t*(24) = 1.946, *p* = .063. Inspection of Fig. 1 shows that in this condition, the PDS group had higher thresholds than the RDS group though there is still considerable overlap in performance between the two groups (backward masking: persistent = 56.2 dB SPL, recovered, 46 dB SPL; notched backward masking: persistent = 50.7 dB SPL, recovered, 42.3 dB SPL. Thus it appears that PDS participants may have poorer auditory processing ability in backward masking conditions than RDS participants.

## 3 Discussion

The backward masking results show that participants who persist in their stutter have poorer backward masking thresholds than those participants who recover from their stutter (the average difference is about 10 dB). Two previous reports of backward masking threshold of people who stutter produced conflicting results. Howell et al. (2000) found backward masking deficits of participants who stutter aged 8–12 compared to fluent controls. Howell and Williams (2004) found no compelling evidence for such differences over a wider age range (there was some evidence that threshold changed at differential rates over age for fluent speakers and speakers who stutter). The current result may resolve the difference in results assuming there were more participants in Howell et al. (2000) who were destined to persist in their stutter. Consistent with this, the ages of the children in Howell et al. (2000) were somewhat younger than in the current study and were at an age at which recovery is still reported (Andrews & Harris, 1964). On the other hand, however, it is not clear why there should have been more children destined to persist in stuttering in the Howell et al. (2000) study than in Howell and Williams (2004).

Higher variability was seen in the speakers who persisted compared with the speakers who recovered from their stuttering. Differences in variability of backward masking performance have also been noted with SLI children compared to controls (Hill, Hogben, & Bishop, 2005; Wright et al., 1997). It has been hypothesized that individuals with SLI may present with similar symptoms though the problem may arise in different individuals due to several different causes (Hill et al., 2005). Poor auditory ability could be one of these causes. If only those individuals with this etiology show poor thresholds, there would then be higher variance in threshold performance across a heterogeneous group of SLI children. A similar argument might be applied to speakers who stutter as it has been proposed that stuttering can arise for many different reasons (Smith, 1999) and this multiplicity of causes could lead to an increase in variance as discussed with SLI children. If this account is true, the current study suggests that a backward masking auditory deficit is one indication of persistent stuttering and that there are others (given that only this group shows high variance in threshold). Moreover, as less variability is observed with the recovered speakers who stutter, they would not appear to be subject to the same spectrum of causes as the persistent speakers.

The findings suggest that an appropriate theoretical account of stuttering to incorporate these findings is one: (a) where an auditory deficit is sufficient, but not necessary, for the disorder to persist (based on the observations about variability), and (b) when auditory deficits are implicated, central auditory processes are involved (only backward masking of the test conditions in this study showed a deficit in the speakers who persist in their stutter over those who recover).

Many contemporary models that address why altered auditory feedback improves the fluency of people who stutter. Different auditory processing loci have been suggested: for instance, Howell (2002) suggests the cerebellum is affected by auditory input, and Alm (2005) proposes that there is a shift from a route involving what he calls the ‘medial’ system (operates automatically and uses the basal ganglia and supplementary motor area) to a ‘lateral’ system (lateral premotor cortex and cerebellum) when altered feedback is switched on. Howell's (2002) EXPLAN theory also addresses why loss of the auditory component does not lead to a cessation of stuttering and why other temporally structured inputs like a flashing light (Kuniszyk-Jozkowiak, Smolka, & Adamczyk, 1996) can affect the fluency of people who stutter. Both of these demonstrate auditory input is not a necessary pre-condition for changes in stuttering behavior.

Over and above the fact that all speakers who stutter (even those who persist) do not have a backward masking deficit, it is unlikely that the deficit is sufficient to precipitate stuttering in those who have this etiology. However, it would appear that hearing measures offer some indication about whether particular children will persist in their stutter. The tests reported here were made at teenage. Tests made with younger children who are then followed up (such as with the younger children in Howell & Williams’, 2004 study) would establish whether the deficit is a sign available early on about whether the children will subsequently persist in the disorder or not for selected children. A restriction in making this test is that testing with Howell and Williams’ (2004) procedure may not be possible with children less than 8 years old. If this problem can be circumvented and an early difference found, backward masking thresholds could then be used in conjunction with other measures, to make clinical decisions about the different group of children.

The results on threshold differences between persistent and recovered participants’ both underline the importance of testing younger children's thresholds, following them up to an age whether it can be decided whether they persist or recover and then examining threshold performance retrospectively. This is the only way in which it can be determined whether the threshold differences are symptomatic of the way stuttering will progress or are a result of deteriorating auditory performance as the disorder persists. This question is important to address because of the practical implications this would have for early diagnosis of persistence of the problem and for theories of the aetiology of the disorder (for example, as to whether stuttering when it starts is in its adult form or whether something changes in late childhood that makes the disorder persist).

## 4 Conclusions

The results show that PDS participants have poorer sensitivity with band-limited backward masking stimuli compared with the RDS participants (a difference of about 10 dB). At present it is not known whether the same results (backward masking) apply at ages closer to onset of the disorder and, if so, whether this could be used as an indicator of prognosis of the disorder.

## Figures and Tables

**Table 1:** Gender, fluency group (PDS^a^/RDS^b^), age when initially assessed and at time of test and SSI-3 scores at these two times for the individual participants numbered by fluency group in column one

ID	Gender	Group^a^^,^^b^	Age at initial assessment (months)	SSI at initial assessment	Age at hearing assessment (months)	SSI at hearing assessment
1	Female	PDS	118	30	163	25
2	Male	PDS	110	34	156	36
3	Male	PDS	168	30	201	30
4	Male	PDS	148	39	196	27
5	Female	PDS	124	28	157	25
6	Male	PDS	102	25	147	29
7	Male	PDS	145	22	174	27
8	Male	PDS	114	40	145	37
9	Male	PDS	119	38	149	33
10	Male	PDS	133	31	162	25
11	Male	PDS	118	31	183	31
12	Female	PDS	114	30	160	31

1	Male	RDS	103	31	184	18
2	Male	RDS	123	26	191	6
3	Male	RDS	98	31	166	18
4	Male	RDS	119	30	176	14
5	Male	RDS	163	37	197	19
6	Male	RDS	119	31	167	18
7	Female	RDS	117	24	164	16
8	Male	RDS	138	23	180	8
9	Female	RDS	143	33	176	19
10	Male	RDS	134	23	166	11
11	Male	RDS	139	25	175	21
12	Male	RDS	113	26	162	9
13	Male	RDS	107	25	147	13
14	Male	RDS	148	22	195	9
15	Male	RDS	112	22	154	8
16	Male	RDS	119	34	155	15
17	Male	RDS	112	22	151	9
18	Male	RDS	142	31	155	22

a PDS stands for persistent developmental stutterer.

b RDS stands for persistent developmental stutterer.

**Table 2:** Speech rating scale questions, with scale endpoints and Boberg and Kully (1994) scale references

Question	Scale endpoints	Boberg and Kully scale references
How would you currently rate your speech?	1 = terrific, 5 = terrible	2
How often are you able to speak fluently without thinking about your speech?	1 = always, 5 = never	6
How much are you stuttering/stammering now compared to before you first saw your therapist/pathologist?	1 = much less, 5 = much more	9
How do you feel about your speech now compared to before you first saw your therapist/pathologist?	1 = much better, 5 = much worse	10
How would you describe your consultation with your therapist/pathologist?	1 = very helpful, 5 = of no help	11
Overall, how much of a problem to you is your stuttering/stammering now, compared to before you first saw the therapist/pathologist?	1 = much less, 5 = much more	12
At this time do you consider yourself a person who stutters/stammers?	1 = definitely not, 5 = definitely yes	14
Do you think you would benefit from seeing the therapist/pathologist again?	1 = definitely not, 5 = definitely yes	3, 4 and 5
